# Instability of Financial Time Series Revealed by Irreversibility Analysis

**DOI:** 10.3390/e27040402

**Published:** 2025-04-09

**Authors:** Youping Fan, Yutong Yang, Zhen Wang, Meng Gao

**Affiliations:** School of Mathematics and Information Sciences, Yantai University, Yantai 264005, China

**Keywords:** financial time series, irreversibility, Kullback–Leibler divergence, sliding window

## Abstract

Since the 2008 global economic crisis, the detection of financial instabilities has garnered extensive research attention, particularly through the application of time-series analysis. In this study, a novel time-series analysis method, integrating the Kullback–Leibler Divergence (KLD) metric with a sliding window technique, is proposed to detect instabilities in time-series data, especially in financial markets. Global financial time series from 2004 to 2022 were analyzed. The raw time series were preprocessed into return rate series and transformed into complex networks using the directed horizontal visibility graph (DHVG) algorithm, effectively preserving temporal variabilities in network topologies. The KLD method was evaluated through both retrospective analysis and real-time monitoring. It successfully identified idiosyncratic incidents in the financial market, correlating them with specific economic events. Compared to traditional metrics (e.g., moments) and econometric methods, KLD demonstrated superior performance in capturing sequence information and detecting anomalies without requiring linear regression models. Although initially designed for financial data, the KLD method is versatile and can be applied to other types of time series as well.

## 1. Introduction

Irreversibility is an important property of nonlinear time series in the real world [1,2,3]. Time series reversibility implies that the original time series exhibits the same joint density distribution as its reversed counterpart [1]. A stationary process X(t) is said to be time-series reversible if, for every *N*, the series $X\left(t_{1}\right),\ldots ,X\left(t_{N}\right)$ and $X\left(t_{N}\right),\ldots ,X\left(t_{1}\right)$ have the same joint probability distributions. Conversely, if this condition does not hold, the time series is considered to be irreversible [1,4,5]. Some previous studies have shown that irreversibility is prevalent in real-world time series, particularly within the socio-economic domain [1,5,6,7,8,9,10,11,12,13].

Kullback and Leibler [14] first introduced the concept of Kullback–Leibler divergence (KLD, also known as relative entropy). KLD evolved from information entropy, which measures the irreversibility of a time series by utilizing the stochastic nature of the continuous time series and inferring the information gain of the statistical model [12,15]. This approach provides a valuable framework for understanding the difference between two probability distributions, making it particularly useful for evaluating how well a model predicts the observed data over time. The divergence quantifies the ‘distance’ between the model and the actual data, which is critical in detecting shifts in financial markets or identifying anomalies in economic behavior. It is also found that KLD is a simple measure of the irreversibility of real-valued smooth random sequences [16,17].

In recent years, Lacasa et al. [18] presented an interesting method in time-series analysis based on graph theory, where the original time series was mapped into a complex network by a visibility graph (VG) algorithm, or it simplified version, a horizontal visibility graph (HVG) algorithm [19]. This method has been particularly effective in detecting nonlinear dependencies and patterns in time-series data, providing a more comprehensive understanding of temporal relationships than traditional methods. Lacasa et al. [20] also proposed a directed version of the horizontal visibility algorithm (DHVG) in 2012 and showed that it was a simple and well-defined tool and uses the visibility algorithm to quantify temporal irreversibility. The DHVG algorithm has gained popularity due to its ability to capture the directionality and asymmetry in the time-series data, offering deeper insights into the processes driving the observed dynamics [20]. By applying KLD to the DHVG graph representation, researchers can quantify the asymmetry and temporal irreversibility in financial time-series data, revealing underlying economic patterns that are often masked in traditional models. Flanagan and Lacasa [21] applied this approach to financial data by adding the idea of coarse-graining.

This paper presents a novel method for analyzing financial stock time series (FSM) based on the time-series irreversibility, measured using the KLD metric, within the framework of the sliding window technique and Monte Carlo tests. The autocorrelations in the financial time series have been considered in constructing the 90% confidence intervals by employing the Monte Carlo method, and then the statistical significance of the empirical KLD can be tested. On the one hand, the retrospective analysis can be implemented within the proposed framework, where the economic crisis periods in the stock indices of various countries can be pinpointed. On the other hand, when new data enter the sliding window, the proposed method can be applied in monitoring abrupt changes in the time series.

The remainder of this paper is structured as follows: In Section 2, we outline the methodology for assessing irreversibility within the context of the sliding window technique. Section 3 introduces the datasets comprising the original financial time series and describes the pre-processing techniques employed in this study. In Section 4, we firstly evaluate the performance of the proposed KLD method from the perspective of retrospective analysis and real-time monitoring. The advantages of the KLD metric are also compared with other moment-based metrics: mean, variance, skewness, and kurtosis. In Section 5, the main findings of this study are summarized and discussed.

## 2. Methods

### 2.1. Visibility Graph, Horizontal Visibility Graph and Directed Horizontal Visibility Graph

The VG algorithm, initially proposed by Lacasa et al. [18], is a set of methods that transform time-series data into network structures based on specific geometric criteria. The main objective of these methods is to convert the information embedded in a time series into an alternative mathematical representation, thereby enabling the application of graph theory tools to analyze the series from a different perspective. This approach helps bridge the gap between nonlinear time-series analysis, dynamical systems, and graph theory [20]. Specifically, the HVG algorithm is a subclass of the VG algorithms that provides a robust framework for this transformation. Let ${\left\{x_{t}\right\}}_{t=1,\ldots ,N}$ represent a real-valued time series with *N* data points. In this method, each data point $x_{t}$ is mapped to a node in the HVG [15]. Two nodes, *i* and *j*, in the graph are considered connected if a horizontal line can be drawn between $x_{i}$ and $x_{j}$ in the time series without intersecting any intermediate data points. That is, for all *n* such that i<n<j, the following condition must be satisfied:

$$x_{i},x_{j}>x_{n},\;\forall n\;|\;i<n<j.$$

**Figure 1:**
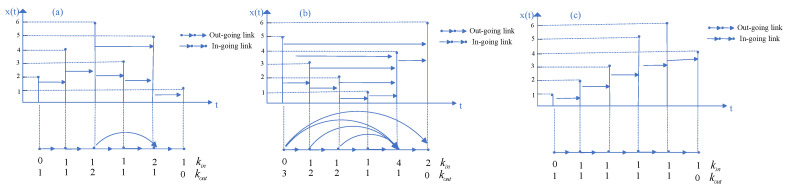
Schematic diagram of directed horizontal visibility graph (DHVG). (**a**) A simple time series $\left\{x_{1},x_{2},x_{3},x_{4},x_{5},x_{6}\right\}=\left\{2,4,6,3,5,1\right\}$ is mapped in a complex network with 6 nodes by the DHVG algorithm. Arrows represent allowed directed visibility between nodes. Consequently, we obtain two degree series: $k_{t}^{\mathrm{in}}=\left\{0,1,1,1,2,1\right\}$ and $k_{t}^{\mathrm{out}}=\left\{1,1,2,1,1,0\right\}$. (**b**,**c**) The original time series were shuffled and became $\left\{x_{1},x_{2},x_{3},x_{4},x_{5},x_{6}\right\}=\left\{5,3,2,1,4,6\right\}$ (a U-shape series) and $\left\{x_{1},x_{2},x_{3},x_{4},x_{5},x_{6}\right\}=\left\{1,2,3,5,6,4\right\}$ (an inversely U-shaped series), respectively. Compared with the original time series, the moments of these three segments of the time series are the same but the KLD values based on the mapped networks are different.

Considering the predefined time arrow, as suggested in previous works [20], the directionality can be explicitly introduced through the use of directed networks or digraphs. This leads to the definition of the DHVG, a directed variant of the HVG. In this directed graph, the degree k(t) of node *t* is split into an ingoing degree, $k_{\mathrm{in}}\left(t\right)$, and an outgoing degree, $k_{\mathrm{out}}\left(t\right)$, such that:

$$k\left(t\right)=k_{\mathrm{in}}\left(t\right)+k_{\mathrm{out}}\left(t\right),$$

where the ingoing degree $k_{\mathrm{in}}\left(t\right)$ is defined as the number of links from node *t* to past nodes (i.e., nodes with $t^{\prime }<t$), and the outgoing degree $k_{\mathrm{out}}\left(t\right)$ represents the number of links from node *t* to future nodes. By introducing this directionality, the DHVG introduces asymmetry into the visibility graph, allowing for a more refined characterization of the temporal dynamics of the time series. The DHVG proposed in [20] is elaborated stepwise below, and Figure 1 presents the many steps of this method:

(1) For a time series $x=\{x_{1},x_{2},\ldots ,x_{N}\}$, we assign each datum of the series to a node in the following horizontal visibility graph.

(2) If $x_{i}>x_{k}$ and $x_{j}>x_{k}$ (∀k,i<k<j), there will be an arrow from $x_{i}$ to $x_{j}$. Then, use $k_{\mathrm{out}}$ to represent the number of arrows out and use $k_{\mathrm{in}}$ to represent the number of arrows in. This condition ensures that only those pairs of nodes that are visible to each other based on the visibility rule will have a directed edge between them. The visibility rule itself can be thought of as a way to capture relationships in the time series by examining the relative heights of values over time.

(3) Calculate $k_{\mathrm{out}}$ and $k_{\mathrm{in}}$, then the probability of each node can be expressed as follows:

(1) $$\begin{array}{c} \begin{array}{cc} p_{\mathrm{out}} & =\frac{k_{\mathrm{out}}}{N};\\ p_{\mathrm{in}} & =\frac{k_{\mathrm{in}}}{N}. \end{array} \end{array}$$

In this context, $p_{\mathrm{out}}$ and $p_{\mathrm{in}}$ represent the normalized out-degree and in-degree of the node, which are useful for understanding the flow of information or connections in the graph. The probabilities indicate how central or influential a given time point is within the network.

Using the segment of the time series presented in Figure 1a as an example, the original data $\left\{x_{1},x_{2},x_{3},x_{4},x_{5},x_{6}\right\}=\left\{2,4,6,3,5,1\right\}$ correspond to 6 nodes in the mapped complex network. The connectedness between any two nodes is determined based on the horizontal and forward “visibility” outlined in step (1). The connectivity among all nodes can be represented by the following adjacency matrix:

$$\left[\begin{array}{cccccc} 0 & 1 & 0 & 0 & 0 & 0\\ 0 & 0 & 1 & 0 & 0 & 0\\ 0 & 0 & 0 & 1 & 1 & 0\\ 0 & 0 & 0 & 0 & 1 & 0\\ 0 & 0 & 0 & 0 & 0 & 1\\ 0 & 0 & 0 & 0 & 0 & 0 \end{array}\right]$$

The summations of all columns of this adjacency matrix produce the in-degree series $k_{t}^{\mathrm{in}}=\left\{0,1,1,1,2,1\right\}$, while the summation of all rows produces the out-degree series $k_{t}^{\mathrm{out}}=\left\{1,1,2,1,1,0\right\}$. Accordingly, the in-degree and out-degree probability distributions are identical.

$$\begin{array}{c} \begin{array}{c} P_{in}\left(k\right)=P_{out}\left(k\right)=\left\{\begin{array}{cc} \frac{1}{6}, & k=0\\ \frac{4}{6}, & k=1\\ \frac{1}{6}, & k=2. \end{array}\right. \end{array} \end{array}$$

The above process of transforming a time series into a complex network with directed links, as well as the calculation of in-degree and out-degree from the adjacency matrix, are implemented within the popular statistical computing environment, specifically in the statcomp package (Version: 0.1.0) [22] and igraph package (Version: 2.1.4) [23].

### 2.2. Kullback–Leibler Divergence (KLD) and Monte Carlo Test

As mentioned in [20], KLD was originally introduced as a measure to distinguish two probability distributions in information theory. In this study, KLD is also used to distinguish probability distributions of the forward and backward appropriate symbolic time series (illustrated in Figure 1). The calculation of KLD is based on the following equation:

(2) $$\begin{array}{c} \begin{array}{c} D[P_{\mathrm{out}}\left(k\right)||P_{\mathrm{in}}\left(k\right)]=\sum_{k}P_{\mathrm{out}}\left(k\right)\log\left(\frac{P_{\mathrm{out}}\left(k\right)}{P_{\mathrm{in}}\left(k\right)}\right) \end{array} \end{array}$$

It is noteworthy that, if the out-degree and in-degree probability distributions of a time series are identical, i.e., $P_{\mathrm{in}}\left(k\right)=P_{\mathrm{out}}\left(k\right)$, then $D[P_{\mathrm{out}}\|P_{\mathrm{in}}]$ is 0; otherwise, it is positive. Furthermore, in the case of $P_{\mathrm{in}}\left(k_{0}\right)=0$, we can directly omit the $k_{0}$ corresponding to the KLD value. It is essential to mention that KLD is associated with the probability of failure when conducting a hypothesis test, or equivalently, it is a measure of “irreversibility”: the greater the distinguishability between $P_{\mathrm{out}}$ and $P_{\mathrm{in}}$, the larger $D[P_{\mathrm{out}}\|P_{\mathrm{in}}]$ becomes, indicating stronger irreversibility of the time series.

In this context, “irreversibility” refers to the degree to which the temporal evolution of a system deviates from symmetry when observed in reverse. A larger KLD value indicates that the system exhibits more pronounced irreversibility, making it more likely to detect significant shifts in the time series that signal abnormal events or transitions.

The sliding window method enables us to observe and compute the behavior of the time series within localized segments, capturing more granular changes and patterns over time. By sliding the window across the entire time series, we can detect shifts that might otherwise be missed in a global analysis. This technique is particularly useful for identifying transient events that might be indicative of a financial crisis or other significant transitions in a market.

**Figure 2:**
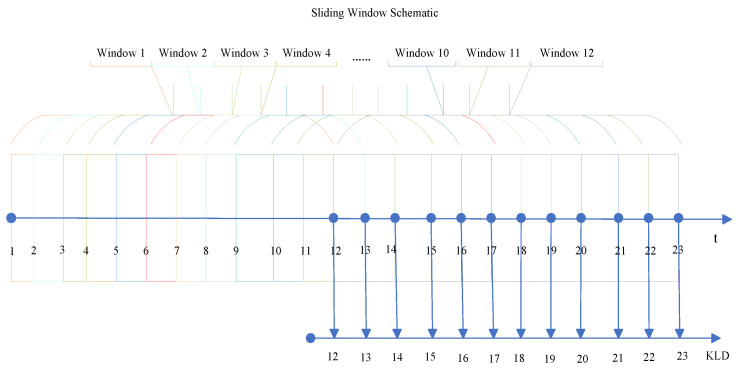
Schematic diagram of KLD statistic within the framework of the sliding window method.

In this study, the KLD is calculated within the framework of the sliding window technique to detect abnormal events in the time series. The sliding window method, also referred to as the rolling window approach, is a prevalent technique in time-series analysis. It involves partitioning the entire time-series data into smaller, overlapping segments or “windows” to analyze specific characteristics or patterns within each window (depicted in Figure 2).

By employing the sliding window approach, each segment of the time series can be treated as an independent observation, allowing us to examine the local dynamics and compare them against the overall distribution of surrogate data. This provides more flexibility and adaptability when detecting abnormal events or changes in the system’s behavior.

The implementation of the proposed method, along with the associated Monte Carlo test, is outlined as follows:

(1) Generate 100 surrogate time series based on the raw time series using the amplitude adjusted Fourier transform (AAFT) method, ensuring that serial correlations are preserved in the surrogate time series. The AAFT method ensures that the surrogate series maintains the same linear structure and statistical properties as the original time series while shuffling the data to destroy any nonlinear dependencies. This helps in generating realistic surrogate data that can be used for hypothesis testing.

(2) Divide both the raw and surrogate time series into moving and sliding windows, and compute the KLD statistic for each sliding window. Each window is analyzed independently to assess the local irreversibility. The KLD statistic is computed for both the raw and surrogate series within each window, providing a dynamic view of the divergence between the forward and backward symbolic representations.

(3) Establish the 90th percentile of KLD values as the threshold to construct a confidence interval based on the 100 surrogate time series. This step ensures that the threshold for detecting abnormal events is derived from the distribution of KLD values obtained from surrogate data. By establishing the 90th percentile, we create a confidence interval that represents typical fluctuations in the surrogate data.

(4) Compare the KLD value of the original time series with the confidence interval. Idiosyncratic events are identified if the actual KLD values exceed the 90% confidence interval. If the actual KLD value from the original time series exceeds the established confidence interval, it signals an anomalous event that is statistically significant and deviates from the expected behavior. These outliers may correspond to significant financial market changes, economic shocks, or other critical events.

The retrospective analysis can be implemented by directly applying the above method to historical time series. For real-time data, a sliding window moves with a small time step to incorporate newly arriving data. When an abnormal value arrives, both the in-degree and out-degree distributions change sharply, resulting in an abrupt change in the KLD value. The statistical significance of the new KLD value can also be tested using the Monte Carlo method.

In this study, we will select two financial time series and conduct retrospective analysis based on historical data. The historical time series will be divided into two parts, with the second part used to illustrate the monitoring of abnormal changes in the financial time series. The accuracy will also be evaluated using the Kappa coefficient (Cohen’s Kappa). All data in the monitoring period are classified into two categories, normal or abnormal values, and then the level of agreement between retrospective analysis and the real-time monitoring can be assessed. The meaning of the Kappa coefficient can be further interpreted as follows. A Kappa value of 1 indicates perfect agreement between the two classifiers, and zero means that the agreement is no better than what would be expected by chance. Negative values indicate that the agreement is worse than what would be expected by chance. In general, values between 0.2 and 0.4 may be considered fair agreement, 0.4 to 0.6 as moderate agreement, 0.6 to 0.8 as substantial agreement, and values above 0.8 as excellent agreement. More details about the Kappa coefficient can be found in [24].

The performance of the KLD in detecting anomalies in financial time series is also compared with other descriptive metrics in statistics, such as the mean, variance, skewness, and kurtosis. Idiosyncratic incidents are respectively identified by KLD and the first four moments based on the Monte Carlo test in each sliding window. Then, the consistency is also compared using the Kappa coefficient.

## 3. Data

**Figure 3:**
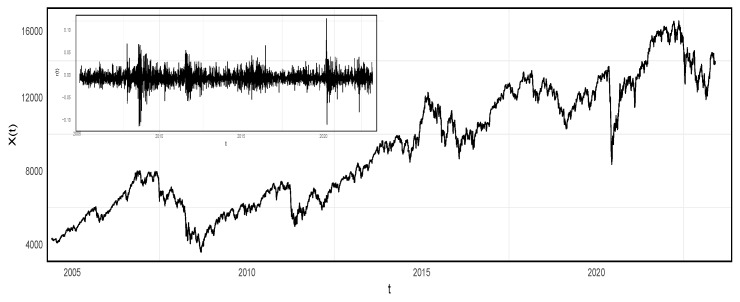
Time series of one stock market index, x(t), and the corresponding return rate series, r(t).

Financial time-series data from 28 countries have been selected to investigate the current state of economic development using the proposed time-series analysis method (data source: https://stock.eastmoney.com/, accessed on 6 May 2024). The countries and their representative stock indices for the period 2004–2022 are listed in Table 1. Prior to applying our method for detecting idiosyncratic incidents, the original time series undergo pre-processing. Firstly, weekends and stock market closures are not considered and are excluded from the time series. Secondly, due to the varying number of holidays per country per year, additional data preprocessing steps are taken. Specifically, in cases where stock data from multiple countries is missing on a particular date, that date is discarded. If the missing data from multiple countries on a given date involves fewer than two countries, the missing values in the raw time series are filled using linear interpolation. Subsequently, the original time series are converted into return rate time series using the formula $r\left(t\right)=\ln\left(\frac{x(t+1)}{x(t)}\right)$ (depicted in Figure 3). In the subsequent section, all analyses will be conducted on the processed return rate time series.

## 4. Results

### 4.1. Clustering and Selection of Financial Time Series

**Figure 4:**
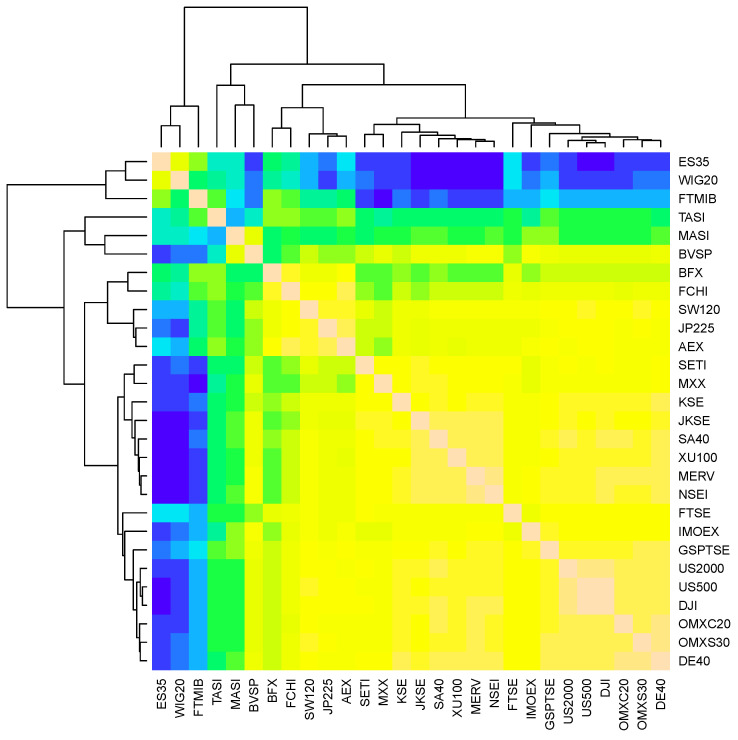
Correlation analysis of international financial stock indices. Financial time series labeled by similar colors (from blue to yellow) are classified into same clusters.

As stated in the Introduction, not all 28 financial time series listed in Table 1 are analyzed one by one in detail, and only a subset of them is selected as representative. Prior to applying the KLD method and Monte Carlo test, the 28 processed return rate time series, derived from the original financial time series, are clustered based on pairwise correlations (as shown in Figure 4). Based on the correlation results, nine return rate time series from Spain, Italy, Saudi Arabia, Morocco, Belgium, Switzerland, Mexico, France, and Russia are selected for detailed analysis. The clustering of the 28 return rate time series can also be implemented based on other correlation metrics. However, we do not intend to illustrate and compare the clustering results with more details or as figures here. The reasons for this are twofold. Firstly, the processed return rate series, which reflect the volatility of the original financial time series, are almost symmetrically distributed around zero (the inlet of Figure 3). Moreover, autocorrelations are not present in these return rate series. It is unnecessary to employ more complicated correlation metrics in the classification process. Secondly, our primary objective in this study is to present the performance of KLD in detecting anomalies in financial time series. The selection of the time series does not qualitatively affect the detection results.

### 4.2. Evaluation of the KLD Method

In this study, the sliding window is configured with a length of 255, which corresponds to all business days in a year, and a moving speed of 25. The configuration of the parameters of the KLD method, such as the length of window size, will be discussed later. Within each window, the corresponding KLD values are computed and the 90% confidence intervals (CI) are estimated based on the Monte Carlo test.

**Figure 5:**
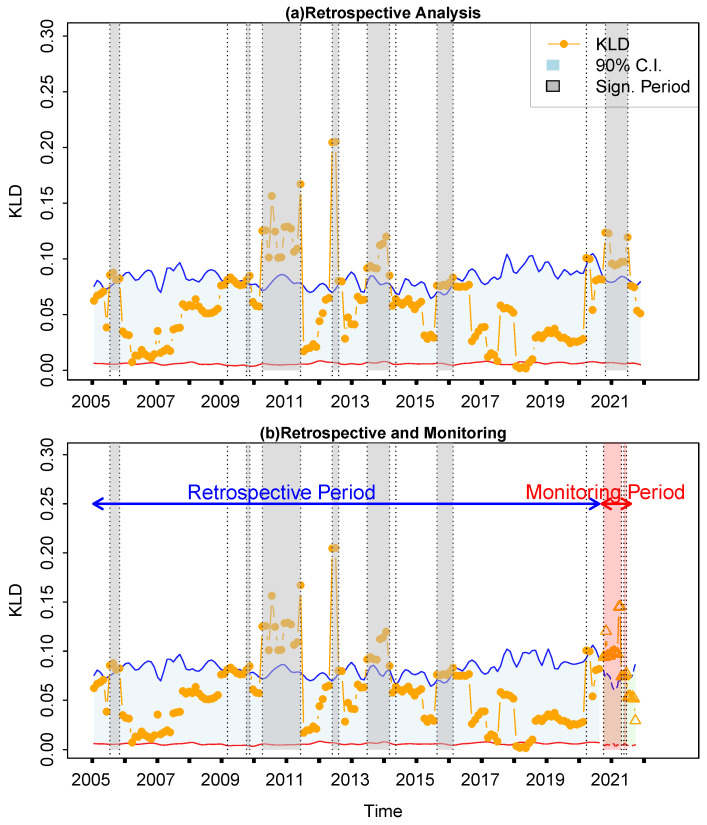
Retrospective analysis and real-time monitoring of financial time series from Spain. Only statistical significant points outside the confidence intervals (marked with the shaded boxes, either in grey or red) are recognized as idiosyncratic incidents and interpreted. (**a**) Retrospective analysis for the whole period from 2004 to 2022. (**b**) Comparison between retrospective analysis and real-time monitoring. Yellow dots represent the KLD series in the retrospective period, while yellow triangles represent the KLD series in the monitoring period. The light grey areas are the 95% confidence intervals, where the upper and lower limits are indicted by the blue and red solid lines.

Firstly, the financial time series from the Spanish markets is selected to illustrate the application of the proposed KLD method for retrospective analysis and real-time monitoring. The whole return rate time series from 2005 to 2022 will be analyzed using the proposed method, so that idiosyncratic incidents can be identified in the retrospective analysis (Figure 5a). In Spain, notable events included the economic boom in 2006, driven by the construction and real estate sectors, which was subsequently followed by the impact of the global financial crisis in 2009. This led to a severe economic downturn characterized by high unemployment rates and banking sector difficulties. During the years 2011–2012, Spain struggled with the sovereign debt crisis in the Eurozone, resulting in austerity measures and further economic contraction. In subsequent years, the economy gradually recovered with structural reforms and external support, leading to improved economic growth in 2014. However, the COVID-19 pandemic in 2021–2022 posed new challenges, particularly impacting sectors like tourism and services, thereby affecting overall economic stability. The time series of return rates is subsequently partitioned into two segments, 2004–2020 and 2021–2022, designated as the retrospective period and the monitoring period, respectively. During the retrospective period, the KLD method was implemented directly. In contrast, during the monitoring period the sliding window advances by one step upon the arrival of each new real-time data point. Figure 5b displays a comparison between the retrospective analysis and real-time monitoring, revealing a satisfactory level of consistency, with a Kappa coefficient of 0.65.

**Figure 6:**
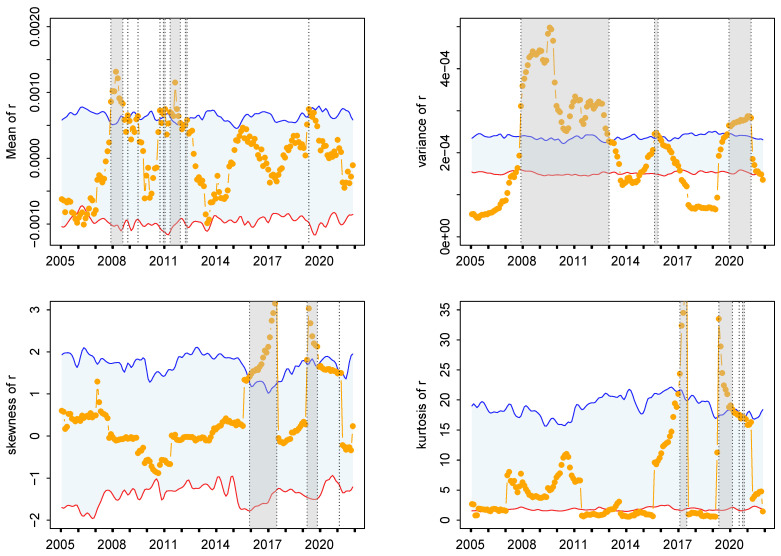
Retrospective analysis for identifying abnormal events from financial time series in Spain: (**top left**) first-order moment, mean; (**top right**) second-order moment, variance; (**bottom left**) third-order moment, skewness; (**bottom right**) fourth-order moment, kurtosis. The symbols have the same meaning as those in Figure 5.

**Figure 7:**
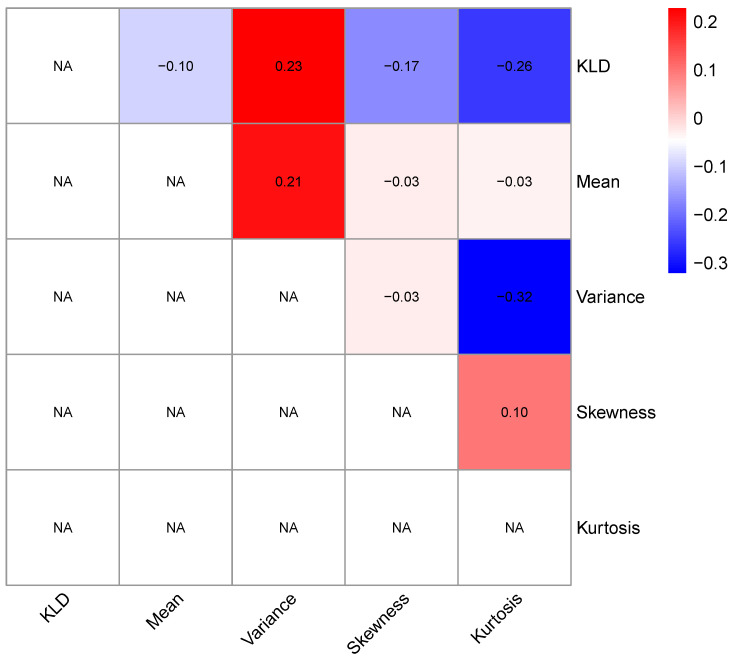
Kappa coefficients among the retrospective analysis results of return rate series in Spain using the KLD metric and four moment metrics. NA means not available.

Secondly, the performance of the proposed KLD method will be compared with that of moment-based metrics: mean, variance, skewness, and kurtosis. The sliding window technique is also applied, and the 90% confidence intervals are constructed accordingly. Figure 6 presents the retrospective analysis results based on the moments. For the first, second, and fourth moments, data points located below the lower bound of the 90% confidence intervals are not considered as abnormal data points. For the first moment (mean), fewer abnormal data points have been detected, especially the idiosyncratic incidents in 2009 and 2020, which have been missed. The widths of the significance periods are also smaller. For the second moment (variance), although more data points have been identified as abnormal, these points are aggregated and considered as single events. For the third moment (skewness), many abnormal data points before 2016 have been missed. For the fourth moment (kurtosis), the identified abnormal data points are almost identical to those identified by the third moment. The consistency between the KLD metrics and the four moment metrics is summarized using the Kappa coefficient, as presented in Figure 7. It can be found that fair agreements exist only between variance and KLD or between variance and mean. The advantage of applying the KLD metric for detecting anomalies can be intuitively illustrated in Figure 1. The three segments of the time series comprise identical data points but vary in their temporal sequences. Consequently, the estimated moment values remain the same, whereas the degree distributions differ. Hence, the KLD metric is capable of detecting the abrupt change in this segment.

**Figure 8:**
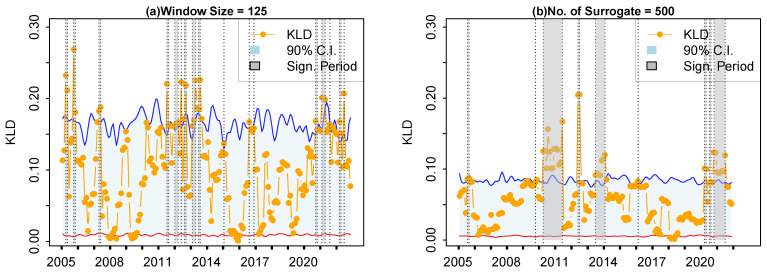
Retrospective analysis based on the KLD metric with different sliding window size or number of surrogate time series. (**a**) Window size is 125. (**b**) Number of surrogate time series is 500. The symbols have the same meaning as those in Figure 5.

Thirdly, the results of retrospective analysis based on the KLD metric with different sliding window sizes or numbers of surrogate time series are presented in Figure 8. In Figure 8a, the sliding window size is 125 (approximately half a year). It can be found that when the sliding window size becomes smaller, more isolated abnormal data points are identified. Thus, single idiosyncratic incidents can be identified as separate incidents. Conversely, independent and consecutive idiosyncratic incidents might be identified as a single event when the sliding window size become too large (not illustrated as figures here). In Figure 8b, the number of surrogate time series is 500. When compared to Figure 5a, the upper boundary of the 90% confidence intervals becomes flatter, but the results of the retrospective analysis do not qualitatively change. We notice that a higher number of surrogate time series is encouraged, although the number 100 is large enough to generate a reliable confidence interval.

**Figure 9:**
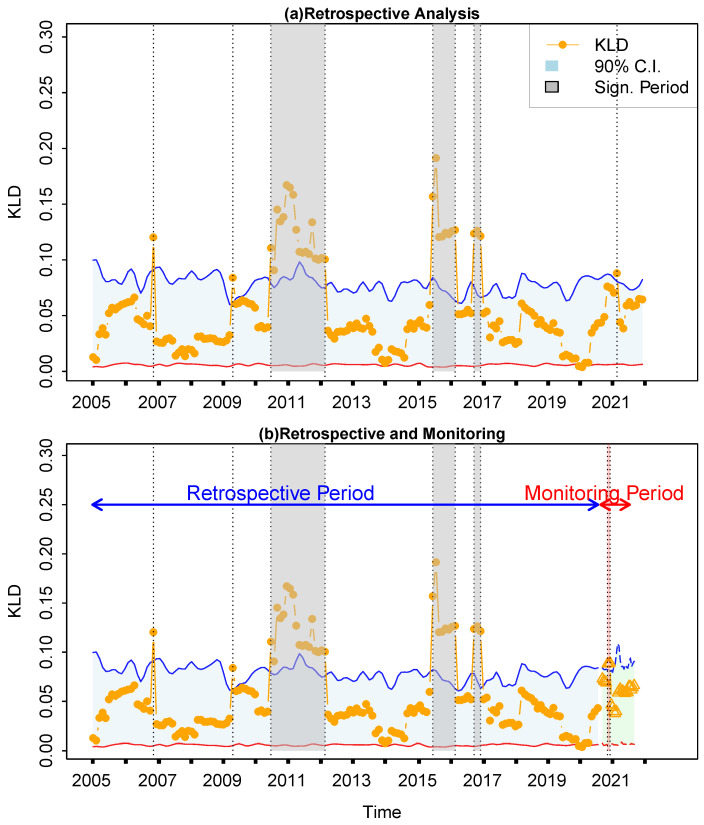
Same as Figure 5, but for Italy. The symbols have the same meaning as those in Figure 5.

Finally, the retrospective analysis and real-time monitoring for another representative financial time series from Italy is presented in Figure 9. From 2006 to 2022, Italy and Spain exhibited remarkable similarities in their economic trends and confronted shared challenges that shaped their economic paths. Similarly, Italy experienced economic fluctuations during the same period. Initial signs of slowdown emerged in 2007, followed by the impact of the European debt crisis in 2010. Between 2011 and 2013, Italy faced economic contraction due to political uncertainties and austerity measures. The banking sector crisis in 2016, coupled with ongoing structural challenges, preceded a moderate economic recovery in 2017. However, economic concerns persisted in 2022, influenced by geopolitical tensions and disruptions in global supply chains. Overall, these events underscore the shared economic challenges and interconnectedness between Spain and Italy, reflecting their common experiences during global economic crises and the efforts made to navigate through periods of economic turmoil and recovery. This example also verifies the proposed KLD method’s capability for real-time monitoring of abrupt changes in financial time series (Figure 9b).

### 4.3. Retrospective Analysis for Other Financial Markets

**Figure 10:**
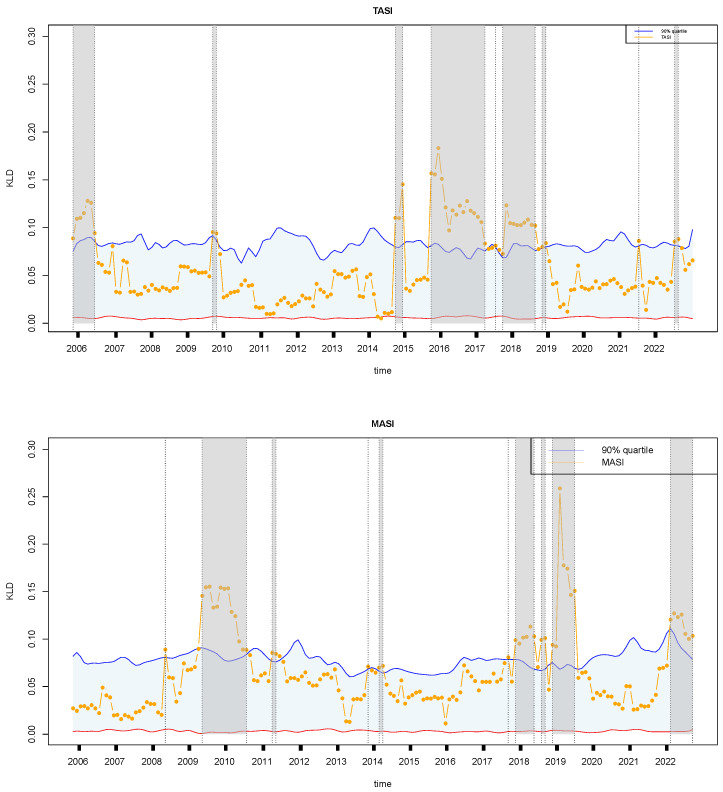
Same as Figure 5a, but for Saudi Arabia (**top**) and Morocco (**bottom**). The symbols have the same meaning as those in Figure 5.

Figure 10 shows the retrospective analysis of financial time series for two Arabian countries, Saudi Arabia and Morocco, which exhibited several similarities while encountering distinct economic events that shaped their trajectories. In Saudi Arabia, pivotal events included a period of robust growth in 2006 fueled by high oil prices and infrastructure projects. By 2010, the country initiated efforts to diversify its economy, responding to the volatility of oil revenues. The sharp decline in oil prices in 2014 necessitated fiscal adjustments and austerity measures to manage economic challenges. Saudi Arabia embarked on a transformative journey with the launch of Vision 2030, a comprehensive reform agenda focusing on economic diversification, privatization, and social development. This initiative, introduced between 2015 and 2017, aimed to reduce reliance on oil revenues and stimulate non-oil sectors. Subsequent years witnessed ongoing economic reforms, including measures to attract foreign investment and enhance competitiveness across industries. However, the COVID-19 pandemic in 2021 posed new economic challenges, impacting global oil demand and necessitating adaptive strategies. Throughout 2022, Saudi Arabia continued its efforts to diversify the economy, navigate market fluctuations, and sustain long-term growth amid evolving global dynamics. Meanwhile, in Mexico, economic events unfolded with their own nuances. The global financial crisis in 2008 reverberated through Mexico’s economy, leading to a period of economic slowdown and challenges across sectors. Subsequent years saw concerted efforts, from 2009 to 2011, to implement stimulus measures aimed at mitigating the recession’s effects through infrastructure investment and social programs. By 2011, Mexico initiated economic reforms focused on enhancing competitiveness and attracting foreign investment, laying the groundwork for sustainable growth. However, the decline in oil prices in 2014 introduced fiscal uncertainties and economic adjustments, influencing the country’s fiscal outlook and growth trajectory. Further developments in 2018 and 2019, including policy changes and trade dynamics such as the renegotiation of NAFTA (now USMCA), shaped Mexico’s economic landscape. In 2022, Mexico continued to navigate economic challenges influenced by global market trends, trade dynamics, and domestic policy measures. In summary, both Saudi Arabia and Mexico demonstrated resilience and adaptability in responding to economic fluctuations, pursuing diversification strategies, and addressing evolving global economic realities during the analyzed period.

**Figure 11:**
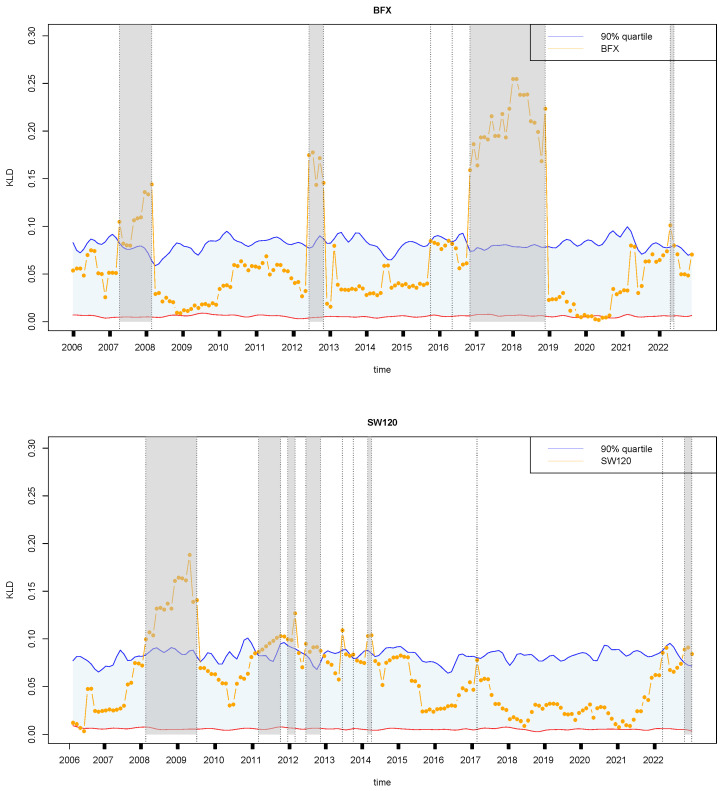
Same as Figure 5a, but for Belgium (**top**) and Switzerland (**bottom**). The symbols have the same meaning as those in Figure 5.

Figure 11 shows the retrospective analysis of financial time series from two European countries, Belgium and Switzerland, during the study period from 2004 to 2022. Both Belgium and Switzerland exhibited notable similarities in their economic journeys, even though they faced periods of economic instability that influenced their unique paths. Belgium, confronted with economic fluctuations, demonstrated resilience and adaptability in the face of external shocks. For example, between 2007 and 2008, Belgium withstood the turbulence of the global financial crisis, enduring economic slowdowns and addressing challenges within its banking sector. However, concerted efforts were made to stabilize the economy, facilitating gradual recovery and enhancing resilience against subsequent economic disruptions. Between 2012 and 2013, Belgium navigated considerable economic uncertainty triggered by the Eurozone debt crisis. The implementation of austerity measures and structural reforms underscored Belgium’s determination to address fiscal pressures and foster economic stability amidst turbulent times. Additionally, in 2016, Belgium’s economic performance was impacted by global trade dynamics, with fluctuating export markets contributing to overall economic instability. Despite these hurdles, Belgium demonstrated resilience and adaptability, focusing on bolstering domestic industries and attracting investments from 2017 to 2019. Nevertheless, the economic landscape remained volatile, particularly in 2022, as Belgium dealt with the lingering effects of the COVID-19 pandemic and shifts in global market dynamics, further underscoring the ongoing economic instability. On the other hand, Switzerland, renowned for its economic resilience, encountered distinct economic events during the same period. From weathering the global financial crisis relatively well in 2008–2010 to managing economic pressures stemming from the Eurozone crisis and currency fluctuations in 2011–2012, Switzerland demonstrated adeptness in navigating economic uncertainties. However, the year 2013 brought challenges as global economic uncertainties impacted sectors such as banking and exports, adding a layer of economic instability. Despite facing such challenges, Switzerland showcased resilience in 2017 amidst geopolitical tensions and currency fluctuations, affecting trade and investment patterns. As Switzerland looks ahead to 2022, ongoing economic developments, including technological advancements and global market shifts, continue to shape its economic outlook, highlighting the ever-present element of economic instability that both Belgium and Switzerland must navigate.

**Figure 12:**
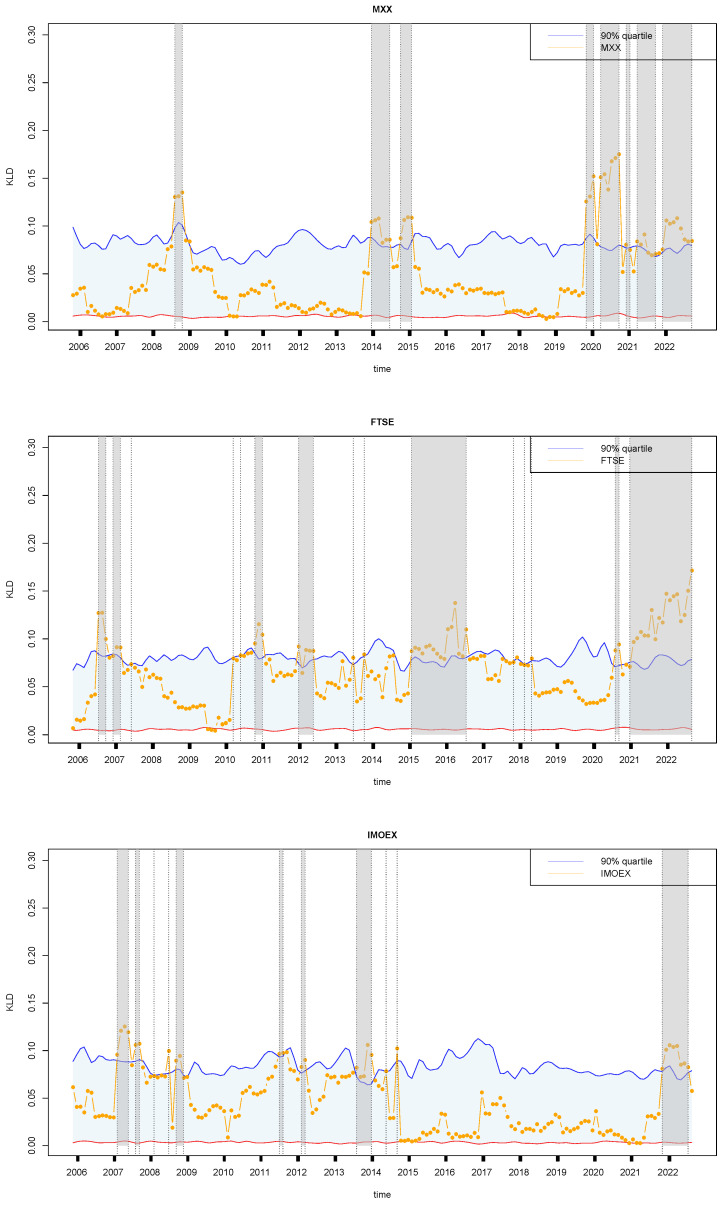
Same as Figure 5a, but for Mexico (**top**), United Kingdom (**middle**), and Russia (**bottom**). The symbols have the same meaning as those in Figure 5.

Figure 12 reveals the similarities and differences in the economic trajectories of Mexico, the United Kingdom, and Russia from 2004 to 2022, highlighting specific events that caused economic fluctuations in each country. Mexico faced significant economic challenges during specific years. In 2008, the country implemented robust economic stimulus measures to counter the severe impacts of the global financial crisis. In 2014, Mexico made strategic adjustments to its tax policies to enhance revenue streams and economic stability. The following year, in 2015, Mexico focused on strengthening financial regulations to safeguard against future financial crises. Subsequently, from 2019 to 2022, Mexico initiated a series of measures aimed at mitigating the economic disruptions caused by the COVID-19 pandemic and navigating through global market turmoil. The United Kingdom experienced notable economic fluctuations and policy responses throughout the research period. In 2006 and 2007, the UK encountered global economic uncertainties, prompting measures to bolster economic resilience. In 2010, the country implemented austerity measures to address the aftermath of the global financial crisis, focusing on fiscal discipline and debt management. The years 2012 and 2015–2017 marked periods of economic pressure related to Brexit negotiations and their implications for trade and investment. Subsequent years from 2018 to 2022 witnessed further economic adjustments as the UK navigated through post-Brexit transitions and evolving global trade dynamics. Russia’s economic landscape was shaped by specific events and policy responses during the study period. In 2007 and 2008, Russia faced economic challenges stemming from oil price volatility and the global financial crisis, leading to strategic policy interventions to stabilize the economy. In 2011, the country implemented targeted economic policies to address geopolitical tensions and enhance economic resilience. Economic reforms were undertaken in 2012 and 2013 to improve competitiveness and address structural challenges. In 2022, Russia continued to navigate economic adjustments amidst geopolitical developments and shifts in global market dynamics, highlighting the ongoing complexities in managing its economy.

**Figure 13:**
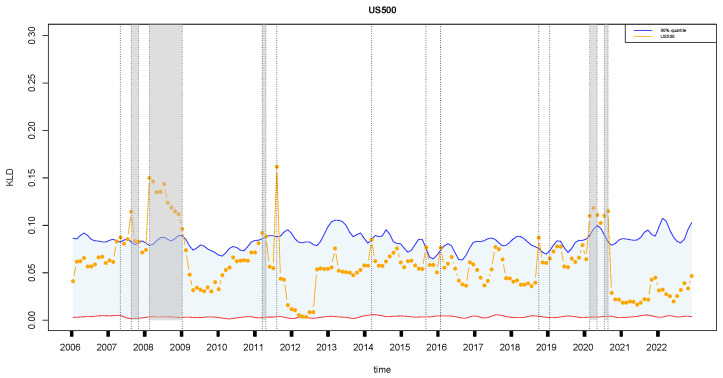

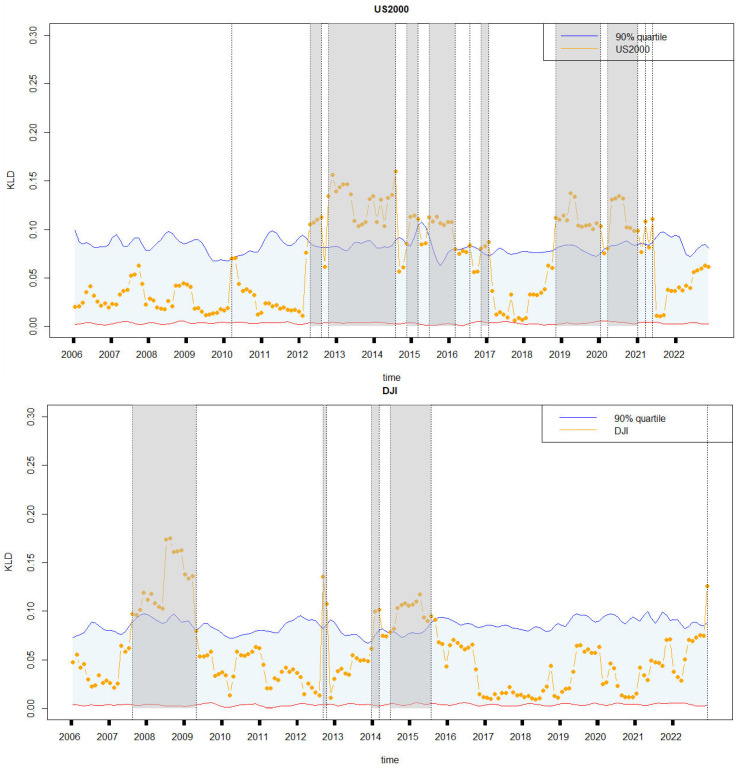
Same as Figure 5a, but for three US financial time series: S&P 500 (**top**), Russell 2000 small cap (**middle**), and Dow Jones Industrial (**bottom**). The symbols have the same meaning as those in Figure 5.

Lastly, three financial time series, S&P 500 (US500), Russell 2000 small cap (US2000), and Dow Jones Industrial Average (DJI), from the United States are analyzed simultaneously to link the financial instabilities and US economy. The development of the United States S&P 500 (US500), Russell 2000 small cap (US2000), and Dow Jones Industrial Average (DJI) from 2006 to 2022 exhibits several similarities in terms of economic trends and instability (Figure 13). Firstly, all three indices experienced significant volatility during the global financial crisis of 2007–2009. The collapse of major financial institutions, the housing market crash, and the credit crunch led to widespread economic turmoil, reflected in sharp declines in stock prices across the board. In 2008, specifically, the S&P 500 (US500) faced instability due to the subprime mortgage crisis, which triggered a cascade of financial distress and uncertainty in the banking sector. The subsequent recession in 2008–2009 further exacerbated the economic challenges, impacting corporate earnings and investor confidence. Similarly, the Russell 2000 small cap (US2000) index witnessed instability in 2010 as a result of concerns about sovereign debt in Europe, particularly in countries like Greece, Portugal, and Spain. These debt crises raised fears of contagion and financial market contagion, leading to heightened volatility in small-cap stocks. From 2012 to 2017, the US2000 index experienced fluctuations due to political uncertainty, including the US government shutdown in 2013 and geopolitical tensions in the Middle East and Ukraine. These events contributed to investor anxiety and market swings, affecting small-cap companies disproportionately. In 2019–2021, the US2000 index faced challenges related to trade tensions between the United States and China, as well as uncertainties surrounding global economic growth and the impact of COVID-19 pandemic-related disruptions on small-cap businesses. On the other hand, the Dow Jones Industrial Average (DJI) demonstrated instability during the 2012 period, driven by concerns about fiscal policy and the fiscal cliff in the United States. Additionally, in 2014 and 2015, the DJI experienced volatility due to fluctuations in oil prices, geopolitical risks, and monetary policy changes by central banks.

## 5. Conclusions and Discussion

KLD is a potent metric that measures irreversibility in time-series analysis. This metric quantifies the dissimilarity between two probability distributions, particularly within the frameworks of probability theory and information theory. In the domain of time-series analysis, KLD is employed to comprehend the complexity and dynamic attributes of temporal data. In this study, KLD was integrated with the sliding window technique to detect instabilities in time series associated with idiosyncratic incidents in financial markets and the progression of economic development. The Monte Carlo method was utilized to establish statistical confidence intervals, with anomalies identified as points lying outside these 90% confidence intervals. In total, we have gathered 28 time series from global financial stock markets spanning from 2004 to 2022. Initially, the raw time series were preprocessed and transformed into return rate time series. Any sudden changes in the original time series can be accurately captured and reflected in the return rate series, whether they are higher or lower values. When the return rate series is transformed into complex networks using the DHVG algorithm, data points with higher or lower values have correspondingly more or fewer links, resulting in higher or lower degree values. Therefore, the temporal variabilities of the original time series can be partly preserved and reflected in the topological structures of the mapped complex networks. The 28 return rate series were first clustered based on correlations, and 12 of them were selected as representatives for further analysis.

The performance of the proposed KLD method was evaluated from two perspectives: retrospective analysis and real-time monitoring. The financial time series from the Spanish markets is firstly selected as an illustration of retrospective analysis. Nearly all the abnormal points detected by the KLD method and classified as idiosyncratic incidents can be effectively correlated with specific economic events that occurred in Spain during the study period. This example also verified that the proposed KLD method can be directly applied for real-time monitoring with a slower moving speed. The KLD metric was also compared with other traditional statistical metrics, such as the moments. When the moments (mean, variance, skewness, and kurtos) were respectively substituted into the proposed method, idiosyncratic incidents were either overlooked or incorrectly identified. As illustrated in Figure 1, we deduced that the KLD metric is capable of capturing sequence information, whereas moment-based metrics fell short in this regard. When the proposed KLD method is compared with traditional anomaly detection methods, such as the “structural changes” detection method in econometrics [25], the proposed KLD method still demonstrates its unique advantages. The traditional structural change test method begins with a linear regression model, where the null hypothesis of “no structural change” implies no alterations in the regression coefficients [26]. Consequently, establishing the linear regression model prior to conducting the test is paramount. In the financial time series analyzed herein, seasonality was not detected in the original time series using seasonal ARIMA models. Furthermore, autocorrelations persisted for an extended period in the original time series but were subdued in the return rate series (not illustrated as figures). With this type of univariate time series, establishing such a linear regression model is hard work. When the proposed KLD method is applied, there is no prerequisite condition and all subsequent calculations are trivial.

Like all sliding window techniques, the proposed method also suffers from the problems of window size sensitivity and edge effect. The selection of an appropriate window size hinges on the length of the target time series and the specific practical context of the research subject. In this study, the sliding window is set to have a length of 255, which is equivalent to the total number of business days in a year. We hypothesized that this window size could effectively encompass the time span during which idiosyncratic incidents occur in the financial market. If the window size is too small, the method will be overly sensitive, whereas a larger window size will render the method insufficiently sensitive. In this study, the edge effect is not a significant issue, and the proposed method is suitable for real-time monitoring purposes. When an abrupt change takes place, both the structure of the mapped complex network and its degree distribution will change correspondingly. Thus, the abrupt change can be detected by the proposed method.

In this study, the proposed method was exclusively applied to financial time series for the purposes of retrospective analysis and real-time monitoring. In reality, this method’s application is not confined solely to financial time series; it can equally be utilized for the detection and prediction of road traffic data, physiological signals, and other intricate systems. This versatility stems from the comprehensive consideration of autocorrelation in the time series during the construction of confidence intervals.

Stochastic volatility has previously been modeled using stochastic differential equations (SDEs) in studies such as [27]. Indeed, SDEs have played a pivotal role in modeling financial time series. The Black–Scholes (BS) model [28] serves as a foundational framework, assuming that asset prices follow geometric Brownian motion (GBM) with constant volatility and interest rates. However, its limitations, including underestimating extreme events and failing to capture volatility clustering or smiles, have spurred significant advancements in the field. To overcome these limitations, Heston [29] introduced stochastic volatility models where volatility itself follows a mean-reverting SDE, thereby allowing for volatility clustering and improving option pricing accuracy. More recently, rough volatility models, inspired by empirical evidence of volatility roughness [30], utilize fractional Brownian motion with Hurst exponents H<0.5 to capture high-frequency volatility dynamics, further refined by the rough Heston model [31]. Additionally, Merton [32] incorporated jumps into SDEs to model sudden price movements, leading to the development of jump-diffusion and exponential Lévy models that accommodate heavy-tailed returns and enhance risk management [33]. Hamilton [34] proposed regime-switching models, where parameters shift between regimes via Markov processes, reflecting structural economic changes. More recently, SDEs have been integrated with machine learning. Sirignano and Cont [35] employed neural networks to identify universal features in price dynamics, while deep-learning methods have expedited high-dimensional SDE calibration [36]. These advancements in SDEs—covering stochastic volatility, jumps, regime-switching, and machine learning—address the limitations of the BS model, offering nuanced representations of the market that enhance derivative pricing, risk assessment, and algorithmic trading. This underscores their enduring relevance in quantitative finance.

However, the stochastic nature of financial time series cannot be adequately represented by the topological parameters of the mapped complex networks. The objective of transforming time series into complex networks is to establish a connection between the statistical properties of the time series and the topological parameters of the networks [18]. To date, mathematical expressions for degree distributions have only been derived for a limited number of time-series types, such as white noise processes and periodic time series [37]. In our prior research, time series were mapped into signed networks, and the autocorrelation within the time series was captured through the degree distribution [38]. We speculate that a clear relationship between time-series fluctuations and topological parameters may be unveiled if appropriate mapping rules are proposed in future studies. The advantage of the proposed method lies in the fact that it does not impose any statistical assumptions on the time series itself. Additionally, autocorrelations are taken into account during the significance test using Mote Carlo methods.

## Figures and Tables

**Table 1:** World Stock Indices. The table below displays stock indices and their abbreviations for selected countries, categorized based on their geographical regions: Countries labeled from (1) to (12) are European countries; (13) represents Saudi Arabia, located in the Middle East; countries labeled from (14) to (16) are in Africa; (17) and (18) represent countries in South America; countries labeled from (19) to (23) are in Asia; (24) to (28) represent countries in North America.

Country	Index
(1) Spain	IBEX 35 INDEX (ES35)
(2) Poland	WIG20 INDEX (WIG20)
(3) Belgium	Belgium BEL20 INDEX (BFX)
(4) France	France CAC40 INDEX (FCHI)
(5) Switzerland	Swiss SWI20 INDEX (SW120)
(6) The Netherlands	Netherlands AEX INDEX (AEX)
(7) England	UK FTSE 100(FTSE)
(8) Russia	MOEX Russia INDEX (IMOEX)
(9) Denmark	OMX20 INDEX (OMX20)
(10) Sweden	OMX Stockholm 30 INDEX (OMX30)
(11) Germany	Germany DAX 30 INDEX (DE40)
(12) Italy	Italy FTSE MIB INDEX (FTMIB)
(13) Saudi Arabia	TASI INDEX (TASI)
(14) Morocco	MASI INDEX (MASI)
(15) South Africa	South Africa 40 INDEX (SA40)
(16) Brazil	Brazil Stock INDEX (BVSP)
(17) Argentina	S&P Merval INDEX (MERV)
(18) Japan	Nikkei 225 INDEX (JP225)
(19) Thailand	Thailand SET INDEX (SETI)
(20) Pakistan	Karachi 100 INDEX (KSE)
(21) Indonesia	Jakarta Composite INDEX (JKSE)
(22) India	India S&P CNX NIFTY INDEX (NSEI)
(23) Turkey	Turkey Istanbul 100 INDEX (XU100)
(24) Mexico	Mexico S&P/BMV IPC INDEX (MXX)
(25) United States	S&P 500 INDEX (US 500)
(26) United States	US 2000 Cash INDEX (US2000)
(27) United States	Dow Jones industrial average INDEX (DJI)
(28) Canada	S&P/TSX Composite INDEX (GSPTSE)

## Data Availability

All the datasets used in this study could be freely accessed from the following website: https://stock.eastmoney.com/ (accessed on 6 May 2024).
